# Smoking status in relation to serum folate and dietary vitamin intake

**DOI:** 10.1186/1617-9625-4-8

**Published:** 2008-09-09

**Authors:** Constantine I Vardavas, Manolis K Linardakis, Christos M Hatzis, Niki Malliaraki, Wim HM Saris, Anthony G Kafatos

**Affiliations:** 1 Department of Social Medicine, Faculty of Medicine, University of Crete, Heraklion, Crete, Greece; 2 Department of Human Biology, Nutrition and Toxicology Research Institute Maastricht, Maastricht University, Maastricht, The Netherlands; 3 University Hospital of Crete, Heraklion, Crete, Greece

## Abstract

**Objective:**

Cigarette smoke itself is an abundant source of free radicals and a major cause of oxidative stress, to which plasma antioxidants function as a vital protective and counterbalancing mechanism. The objective of this study was to investigate into the relationship between smoking status and serum and dietary micronutrient concentrations.

**Design:**

Cross-sectional study

**Subjects – Setting:**

502 farmers from the Valley of Messara in Crete were randomly selected and examined. Complete three-day and 24-hr recall questionnaires were collected along with anthropometrical, physical activity and clinical data from all participating subjects.

**Results:**

After adjusting for age, gender and number of fasting days adhered to per year, current smokers were found to have a lower dietary intake of vitamin C (112.1 mg vs. 136.4 mg, *p *= 0.03), fibre (16.6 g vs. 19.1 g, *p *= 0.006) and fruits and vegetables (339 g vs. 412 g, *p *= 0.014), while dietary vitamin B_1 _intake was found to be higher (1.7 mg vs. 1.4 mg, *p *= 0.02) in comparison to non/ex smokers. Dietary intake of meat, folate and vitami A, E, B_2_, B_6 _and B_12 _did not differ between the groups. Controlling age, gender, fasting days and dietary micronutrient intake, serum folate levels were found to be lower among smokers (geometric mean 15.3 nmol/L vs. 17.7 nmol/L, *p *= 0.023), while serum iron and vitamin B_12 _levels were not affected by smoking status.

**Conclusion:**

Current smoking status affects dietary nutrient intake as well as plasma folate levels. The above coherence between antioxidant depletion and reduced antioxidant intake may predispose smokers to the premature development of tobacco related mortality and morbidity.

## Introduction

Tobacco consumption is one of the worlds leading causes of preventable death. It had been estimated that in the year 2000 almost 5 million premature deaths globally were attributed to smoking, with cardiovascular disease, chronic obstructive pulmonary disease and lung cancer the leading causes of mortality and disability [1]. Both active and passive smoking have been related to the development of cancer and cardiovascular disease with tobacco's plethora of carcinogenic and volatile chemicals responsible for its negative effects on human health [2].

Cigarette smoke itself is an abundant source of free radicals that promote oxidative stress by both the direct delivery of radicals and their endogenous generation, via the activation of inflammatory cells [3]. It has been estimated that in one puff of a cigarette, the gas phase of the smoke exposes the smoker to greater than 10^15 ^free radicals [4]. This oxidative stress acts as a significant risk factor for chronic disease development, such as atherosclerosis and carcinogenesis.

A vital line of human defense against environmental and dietary oxidant intake are the endogenous radical scavengers and the serum antioxidants in the form of dietary micronutrients. Due to the fact that antioxidants are potential pro-oxidants, possible lower concentrations of antioxidants in smokers in comparison to non-smokers may be a defensive adaptation to the pro-inflammatory environment in smoker's tissues [5]. As viewed in the literature, smokers have lower circulating concentrations of a number of micronutrients, such as ascorbic acid, a-carotene, b-carotene and cryptoxanthin. Although it is possible that differences in dietary intakes could potentially account for these disparities, few studies having controlled for differences in nutrient intake. In research study planning and design it is imperative that these differences are accounted for since smokers are generally known to adhere to a less healthy diet, consume fewer food items rich in fibre, antioxidants and phytochemicals and tend to prefer a meat/alcohol dietary pattern in comparison to non-smokers [6-10].

Taking the above into account, the purpose of this study was to investigate into the relationship between smoking status and a) dietary vitamin, folate, fiber, fruit and vegetable intake and b) serum folate, iron and vitamin B_12 _concentrations.

## Methods

### Participant sampling procedure

The questionnaire and biological sample database for this study used the framework of the existing MESSARA cross sectional study. The purpose of the MESSARA cross sectional study was to estimate the prevalence of cancer, cardiovascular and chronic disease and their risk factors among the farming population of the valley of Messara in Southern Crete, and for this purpose extensive questionnaire data and biological samples were stored.

Participants were randomly selected by using the six municipalities's voting lists using a random stratified sampling methodology, with the number of participants in each of the 24 villages selected according to the villages total farming population. A participant was regarded as a farmer if his main occupation was farming and/or sheep breeding, and was concurrently insured by the farmer's social insurance fund. Out of the 662 people randomly selected, 599 accepted to participate (90,5%) out of which 504 (84%) were finally examined with 44% (n = 223) of the participants' male and 56% (n = 281) of female gender respectively (age 18–79). The mean age (± SD) of males was 49.1 (± 12.7) in comparison to 49.2 (± 12.4) for females.

All examinations were conducted during March-May of 2005, by a trained research group of medical doctors, nurses and dieticians, after the population was informed about the study and written consent was given. Approval to conduct the survey was obtained from the University of Crete Ethics committie, while funding was granted by the prefecture of Heraklion's governing authority.

### Dietary Survey

Dietary intake was assessed using a three-day dietary record questionnaire that was handed to the study participants by the local council members of each village, prior to the examination day of the study (n = 251). Written and oral explanations were also provided. Where the three-day dietary record questionnaires were not returned, a trained dietician completed a 24-hour dietary recall questionnaire on the day of examination (n = 249). To re-ensure the representatives between the 35 compounds of the three-day dietary record questionnaires and the 24-hr dietary recall questionnaires, statistical methods were used. The differences were not significant, except for vitamin A intake. Detailed descriptions of all foods, beverages and supplements consumed during the three-day or 24-hour period before the interview, including the quantity, cooking method and brand names were recorded. Dietary records and fasting habits were available from 500 participants. Food quantities were assessed by the use of household measures and color food-model photographs. Nutrient contents were analyzed according to the food database developed at the Department of Social Medicine of the University of Crete in 1998 and updated in 2000 [11]. The database includes about 500 foods, both single and composite. The macro- and micro-nutrient composition of about 20 foods has been chemically determined at Wageningen Agricultural University. The fatty acid content of 105 fat-containing foods was determined at the TNO Nutrition and Food Research Institute (The Netherlands) during 1997. For the remainder of fat-containing foods, the fatty acid analyses were drawn from the analyses available within the European "trans fatty acid research project" database (TTDB, version 1.2) developed at the TNO Nutrition and Food Research Institute between 1995 and 1997. For the foods whose composition was not chemically determined, values from the US Department of Agriculture database v11.1 were used. Recipe calculations were used for composite Greek foods with the ingredients being weighed prior to and also following cooking. For means of comparison with other studies the RDA for vitamins designated by a designated US food and Nutrition board was used [12]. Orthodox Christian church fasting days as a component of the Mediterranean diet of Crete play an important role in the dietary intake of the population and therefore were also taken into consideration during the dietary assessment [13].

### Laboratory measurements

Early morning, venous blood samples were drawn from 457 participants, for biochemical screening tests, following a 12-hour overnight fast. The blood samples were transferred to the Nutritional Research Laboratory of the University of Crete in cases containing ice packs, so as to maintain a temperature of 3–4°C. Quantitative determination of folate and vitamin B12 in serum was performed by competitive immunoassay using direct chemiluminescent technology. (Automated ADVIA Centaur system, BAYER). Serum iron levels were calculated for all 457 participants while funding for 265 randomly selected folate samples was available. 261 vitamin B12 serum levels were calculated, as 4 were lost during the lab analysis.

### Health assessment

Smokers were classified as those who stated that they smoked more than one cigarette over the past three consecutive months. Ex-smokers were defined as those who had not been smoking for the last six consecutive months, and non-smokers as those who did not fall in any of the two previous groups. During the analysis and due to the fact that the body's antioxidant capacity can replenish quickly during periods of high dietary antioxidant, folate and vitamin intake, both ex smokers and non-smokers were grouped together. Body mass Index (BMI) was calculated as body weight in kilograms (kg) divided by the square of the body height in meters (m^2^). Subjects were weighed without shoes, in their underwear or light clothing with an accuracy of ± 100 g, while body height was measured without shoes to the nearest 0.1 cm. Physical activity level was calculated using a weighted weekday and weekend physical activity questionnaire validated and used in a previous study in Crete [14]. Complete data on BMI and physical activity levels were available for 498 participants.

### Statistical analysis

The statistical package SPSS 15.0 was used to perform the analysis. Student t-tests and chi-squared tests (χ^2^) were used during comparisons. Log10 transformed values were used due to abnormality of serum folate, iron and vitamin 12 serum values while geometric means with 95% confidence intervals were presented. Analysis of covariance was performed to eliminate possible confounding factors such as age, gender and number of fasting days in relation to smoking status. Chi squared tests (χ^2^) were also used to compare the frequency of inadequate food and nutrient intake between smokers and non/ex smokers. Statistical significance was noted as *p *< 0.05.

## Results

Smoking prevalence was estimated at 29 % with 48.2 % of male participants and 13.3% of female participants daily smokers. Average years of smoking differed according to gender (male: 28 ± 11 vs. female: 14 ± 7, *p *< 0.001) as also the number of cigarettes smoked daily (male: 31 ± 16 vs. female: 16 ± 11 *p *< 0.001) (Table 1).

**Table 1:** Descriptive and nutritional related characteristics of the study sample in relation to smoking status.

		**Smokers** % (n)	**Ex/Non smokers** % (n)	***P*-value**
**Gender**	**Male**	48.2 (109)	51.8 (117)	**<0.001 **^**1**^
	**Female**	13.3 (37)	86.7 (241)	
**Age ***in years*	**18 – 39**	39.7 (48)	60.3 (73)	**<0.001**
	**40 – 59**	30.6 (81)	69.4 (184)	
	**60 – 80**	13.6 (16)	86.4 (102)	
**Fasting ***in days per year*^3^	**0**	32.2 (47)	19.7 (70)	**<0.001**
	**1 – 100**	58.2 (85)	48.3 (172)	
	**101 – 180**	8.3 (12)	32.0 (114)	

		**Mean **± **SD (n)**	**Mean **± **SD (n)**	
**Body Mass Index**^**2**^		29.6 ± 0.4 (144)	30.2 ± 0.3 (354)	0.286
**Energy intake ***in kcal*^2^		2121 ± 57 (142)	2075 ± 35 (353)	0.507
**Physical Activity Level**^**2**^		2.40 ± 0.06 (144)	2.34 ± 0.04 (354)	0.399

1. Values are % (n). Chi-square test was used χ^2^.

2. Values are Mean ± standard error (n). Analysis of Covariance: gender, age and fasting days were used as covariates.

3. Fasting days per year.

Regarding obesity indexes and physical activity, current smoking status vs. non/ex smoking was not found to effect BMI (29.6 vs. 30.2, *p *= 0.286), daily energy intake (2121 vs. 2075, *p *= 0.5) and the level of physical activity performed (2.4 vs. 2.34, *p *= 0.399) when gender, age and days of Orthodox church fasting per year were taken into account.

The relationship between smoking status and dietary nutrient, fruit and fibre intake as is shown in Table 2. Smokers were found to have a lower daily intake of Vitamin C (112.1 mg vs 136.4 mg, *p *= 0.03), dietary fibre (16.6 mg vs. 19.1 mg, *p *= 0.006) and fruits and vegetables (339 g vs. 412 g, *p *= 0.014) in comparison to non-smokers, while dietary vitamin B_1 _intake was found to be higher among smokers (1.7 mg vs. 1.4 mg, *p *= 0.02). Daily dietary intake of vitamin E although higher among non smokers did not reach statistical significance (*p *= 0.056) while no differences between smokers and non-smokers, were found regarding folate and vitamin A, B_2_, B_6 _and B_12 _intake.

**Table 2:** Daily dietary nutrient, fruit and fibre intake in relation to current smoking status.

	**Smokers **[n= 142]		**Ex/Non smokers **[n = 353]		
	*Daily intake in Weight*	*SD*	*Daily intake in Weight*	*SD*	***P*-value**
**Folate **(μg)	210.1	10.8	230.1	6.6	0.130
**Vitamin A **(μg)	1067	109	1153	66	0.518
**Vitamin E **(mg)	11.3	0.5	12.5	0.3	0.056
**Vitamin C **(mg)	112.1	9.1	136.4	5.5	**0.030**
**Vitamin B**_**1 **_(mg)	1.7	0.1	1.4	0.1	**0.020**
**Vitamin B**_**2 **_(mg)	1.7	0.1	1.8	0.1	0.745
**Vitamin B**_**6 **_(mg)	1.6	0.1	1.6	0.1	0.709
**Vitamin B**_**12 **_(μg)	4.3	0.7	4.6	0.5	0.728
**Fibre **(g)	16.6	0.7	19.1	0.4	**0.006**
**Fruits and Vegetables **(g)	339	24	412	15	**0.014**
**Meat **(g)	88	9.0	90	6.0	0.915

As for serum concentrations (Table 3), folate was found to be statistically significantly higher among non-smokers (17.7 nmol/L vs. 15.3 nmol/L, *p *= 0.023), while iron and vitamin B_12 _serum concentrations did not differ according to smoking status when the participants' dietary intake, gender, age and number of fasting days per year were taken into account.

**Table 3:** Serum folate, iron and vitamin B_12 _concentrations in relation to smoking status

	**Smokers**^**1**^ Geometric Mean (95% Confidence Interval) [N]	**Ex/Non smokers**^**1**^ Geometric Mean (95% Confidence Interval) [N]	***P*-value**
**Serum Folate**^**2 **^(nmol/L)	15.3 (13.8–16.9) [77]	17.7 (16.6–18.9) [188]	**0.023**
**Serum Iron**^**2 **^(μg/dl)	87 (76–99) [131]	80 (73–86) [326]	0.276
**Serum**^**2 **^**Vitamin B**_**12 **_(pg/ml)	354 (325–386) [77]	341 (324–360) [184]	0.495

1. Values are Geometric Mean (95% Confidence Interval) [N].

2. Analysis of covariance (gender, age and the number of fasting days were used as covariates).

Table 4 depicts the investigation into the relationship between smoking status and severely inadequate dietary nutrient intake (designated at less than 67% of the RDA). According to this cut-off, almost 80% of smokers were found to receive less than 2/3 of the RDA for folate, while vitamin deficiencies were also noted in almost 40% of smokers for other micronutrients such as vitamin A, E and C to which almost 40% of smokers. On the other hand, the percentage of the population with B_1_, B_6 _and B_12 _deficiencies was statistically significantly higher among non-smokers in comparison to active smokers (*p *= 0.013, *p *= 0.032 and *p *< 0.001 respectively.) while non-smokers were found to have higher but non significant folate, vitamins A, E, C as well as fiber, fruit and vegetable intake.

**Table 4:** Severely inadequate food and nutrient intake in relation to current smoking status.

	**Smokers** **% (n)**	**Ex/Non smokers** **% (n)**	***P*-value**
**Folate** (<400 μg/day)	94.4 (133)	92.1 (327)	0.371
**Folate** (<268 μg/day)	76.2 (109)	70.7 (251)	0.213
**Vitamin A** (<67% of RDA)	49.7 (71)	42.3 (150)	0.133
**Vitamin E** (<67% of RDA)	39.9 (57)	35.8 (127)	0.393
**Vitamin C** (<67% of RDA)	24.5 (35)	18.0 (64)	0.103
**Vitamin B**_**1**_ (<67% of RDA)	4.9 (7)	12.4 (44)	**0.013**
**Vitamin B**_**2**_ (<67% of RDA)	9.1 (13)	14.1 (50)	0.129
**Vitamin B**_**6**_ (<67% of RDA)	11.2 (16)	19.2 (68)	**0.032**
**Vitamin B**_**12**_ (<67% of RDA)	21.1 (30)	42.5 (148)	**<0.001**
**Fibre** (<15 g)	39.2 (56)	36.3 (129)	0.555
**Fruits and Vegetables** (<67 % of RDA)	36.8 (49)	29.9 (103)	0.142

1. Chi squared test (X^2^).

2. Severely inadequate food and nutrient intake was defined at <67% of the RDA.

## Discussion

Current smoking status was strongly related to dietary patterns, with smokers found to have a lower dietary fibre, fruit and vegetable intake in comparison to non-smokers. Smoking status also affected serum folate concentrations when corrected for dietary intake, while serum iron and serum vitamin B_12 _levels were not associated with current smoking status.

### Smoking among Cretan farmers

As hypothesized, smoking prevalence among the farmers of Crete was elevated, especially among male participants. The 48.2% of male smokers is very similar to the 51% found in urban Athens, while the percentage of female smokers differs substantially according to place of residence with lower smoking rates found among female farmers (13.3% vs 39%) [15]. This could be attributed to the fact that Greek women of higher socio-economic status are more likely to smoke than the less educated or those of lower income while women in rural areas most likely also smoke less, due to the existing traditional culture that regards female smoking taboo [16]. The elevated smoking prevalence as noted above, is a major risk factor for the development of cardiovascular disease and cancer, acting in coherence with the inadequate dietary intake of a number of vitamins, fibre, fruit and vegetables and concurrent depletion of available biological antioxidant deposits due to their smoking habits.

### Smoking status and vitamin deficiencies

Folate and vitamins B_6 _and B_12 _are involved in the regulation of homocysteine, and elevation of homocysteine has been shown to be an independent risk factor for coronary heart disease [17]. Chemical components found in tobacco smoke interact with the above and transform them into inactive compounds reducing their active concentration in biological fluids and possibly alter the ability of the cell to store and metabolise folate [18]. The lower serum folate levels found in our study most likely follow the mentioned mechanism, and other studies have confirmed the finding [19,20].

Regarding B_12 _serum levels, we found that smokers had higher vitamin B_12 _levels in comparison to non-smokers even though the difference was not statistically significant. The existing literature is vague regarding the relationship between smoking and serum cobalamin levels (vitamin B_12_). Two studies investigating into smoking during pregnancy and vitamin status suggest that there may be a dose related relationship between smoking and the metabolism of vitamins B_6 _and B_12 _while a third study (among men only) found significantly higher B_12 _levels among smokers (465 pmol/l vs 314 pmol/l) [21-24]. Although it has been stated, that elevated B_12 _levels among smokers might be attributed to a higher meat consumption, (since smokers are more likely to choose meat instead of fruit and vegetables), both smokers and non smokers of our study population were found to have a similar dietary intake of B_12 _and meat.

Current smoking was not found to effect mean plasma concentrations of iron, a finding that is similar to previous researchers results, that state that although serum ferritin is related to smoking status iron levels are not, especially among men between 40–60 years old [25,26]. BMI, energy intake and level of physical activity were also not found to differ significantly according to smoking status, factors that could be influenced by the fact that the study population is in rural Crete.

### Vitamin deficiencies in smokers and non-smokers among farmers from Crete

The traditional Mediterranean diet of Crete is renowned for its very high consumption of olive oil, vegetables, legumes, fruit, fish, whole-wheat cereals and a moderate consumption of dairy products and meat. Although it is possible that urban populations of Crete may not adhere that strongly to the traditional Mediterranean diet any more, those in rural areas and especially those middle aged or elderly are likely to adhere to the diet rich in antioxidants that historically granted them health and long life expectancy. An important source of the populations antioxidant intake are fruit, vegetables and wildly grown edible greens that are collected and eaten fresh or cooked in pies. Studies have shown that the wild Cretan greens are rich sources of vitamin C, K, E and carotenoids and are capable of significantly contributing to the antioxidant buffer and the RDA needs of the population [27,28].

Both smokers and non-smokers of our study population were found to have a lower folate intake than the recommended 400 μg/day. A study performed in another Mediterranean cohort, in Spain, found that folate intakes of smokers was significantly lower than that of non smokers, as did the INTERMAP study [29,30]. We too found folate intake of smokers to be lower, although not significantly. The INTERMAP study also reported null differences in B_12 _intake between smokers and non-smokers to which our data agree. A US population based study; the CSFII, showed that smokers had a significantly lower dietary intake of vitamins C and D in comparison to non-smokers, a finding that our study does not replicate [31]. It is possible though that the much higher vitamin intake of both smokers and non-smokers in Crete may be due to some degree of adherence to the Mediterranean diet of Crete.

In general and irrespective of smoking status, a relative large proportion of the farming population of Messara has a number of alarming deficiencies in vitamin intake as well as daily fiber, fruit and vegetable consumption, a factor that would affect their predisposition to chronic disease and cancer [32-34]. The fact that almost 40% of the population consumes less than 67% of the RDA for fruit and vegetables might be due to the possibility that the population, or a subgroup of it, may not adhere to the traditional Mediterranean diet of Crete, further studies are needed to have a detailed analysis of this hypothesis. In line with the above, the farming population of the Messara valley could greatly benefit from an educational intervention programme that would stress the necessity of smoking prevention and cessation as also the benefits of adhering to a Mediterranean style diet towards the prevention of future disease development.

In total, the statistical data on the relationship between smoking status and dietary habits as well as plasma levels of folate provide strong evidence that cigarette smoking could be direclty responsible for lowering plasma folate levels and may have a significant impact on smokers' dietary habits and nutrient intake. The above coherence between antioxidant depletion and reduced antioxidant intake has a further impact on the health status of smokers further predisposing them to the premature development of tobacco related death and disease.

## Competing interests

The authors declare that they have no competing interests.

## Authors' contributions

CIV helped design the study, collected data and was the main author of the manuscript, MKL helped design the study, performed the statistical analysis and helped to draft the manuscript, CMH collected data and helped with the study programming, NM performed the sample analysis and helped to draft the manuscript, WHMS participated in data interpretation and helped in drafting the manuscript and AK conceived the study, helped with its design and manuscript preparation. All authors read and approved the final manuscript.
